# Oleuropein improves insulin resistance in skeletal muscle by promoting the translocation of GLUT4

**DOI:** 10.3164/jcbn.16-120

**Published:** 2017-10-03

**Authors:** Yoko Fujiwara, Chisato Tsukahara, Naoe Ikeda, Yasuko Sone, Tomoko Ishikawa, Ikuyo Ichi, Taisuke Koike, Yoshinori Aoki

**Affiliations:** 1 Food and Nutritional Sciences, Graduate Course of Humanities and Sciences, Ochanomizu University, 2-1-1 Otsuka, Bunkyo-ku, Tokyo 112-8601, Japan; 2 Institute for Human Life Innovation, Ochanomizu University, 2-1-1 Otsuka, Bunkyo-ku, Tokyo 112-8601, Japan; 3 Departments of Health and Nutrition, Takasaki University of Health and Welfare, 37-1 Nakaorui-machi, Takasaki-shi, Gunma 370-0033, Japan; 4 Eisai Food & Chemical Co., Ltd., 2-13-10 Nihonbashi, Chuo-ku, Tokyo 103-0027, Japan

**Keywords:** oleuropein, C2C12, insulin resistance, GLUT4, AMPK

## Abstract

As the beneficial effects of the Mediterranean diet on human health are well established, the phenolic compounds in olive oil have been gaining interest. Oleuropein, a major phenolic compound in olives, is known to reduce the blood glucose levels in alloxan-induced diabetic rats and rabbits, however, its effect on type 2 diabetes caused by obesity is not clear. The purpose of this study is clarifying the effect of oleuropein on the glucose tolerance in skeletal muscle under the condition of lipotoxicity caused by type 2 diabetes. Oleuropein enhanced glucose uptake in C2C12 cells without insulin. Translocation of glucose transporter 4 (GLUT4) into the cell membrane was promoted by activation of adenosine monophosphate-activated protein kinase (AMPK) but not protein kinase B (Akt). Physiological concentration of oleuropein (10 µM) was sufficient to express beneficial effects on C2C12 cells. Oleuropein prevented palmitic acid-induced myocellular insulin resistance. Furthermore, in gastrocnemius muscles of mice fed a high fat diet, oleuropein also induced the GLUT4 localization into cell membrane. These results suggest the possibility of oleuropein to be effective for type 2 diabetes by reducing insulin resistance in skeletal muscles.

## Introduction

The increase in lifestyle-related diseases, especially type 2 diabetes, caused by obesity has become a worldwide problem, even in developing countries. The beneficial effects of the Mediterranean diet on human health are well established from many epidemiological studies.^(1–4)^ Consequently, the phenolic compounds in olive oil, a major source of dietary fat in the Mediterranean diet, have been gaining interest.^(5–7)^

**Fig. 1:**
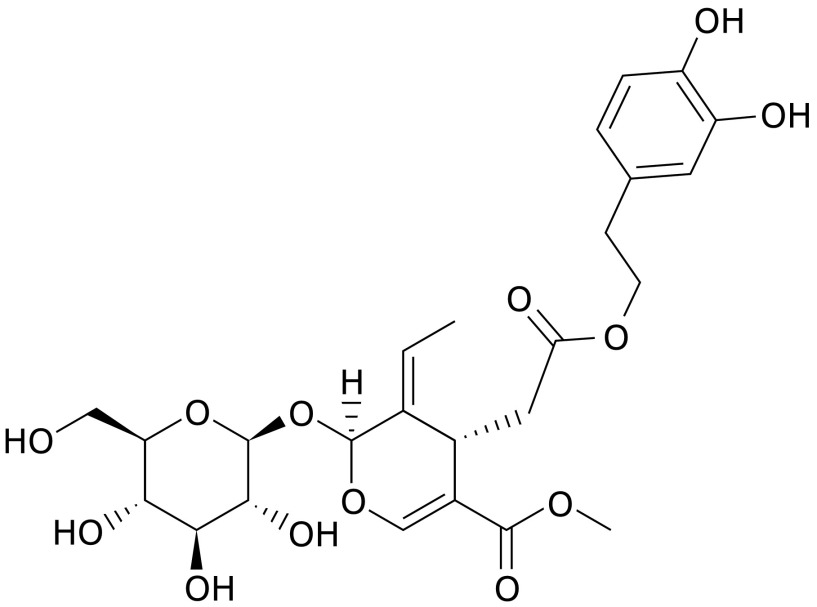
Structure of oleuropein.

Oleuropein (Fig. 1), a nontoxic secoiridoid compound derived from olives and olive oil, is responsible for their typical bitter and pungent aroma.^(8,9)^ Its biochemical functions, including antimicrobial,^(9,10)^ antioxidative,^(6,11,12)^ and anticancer activities^(13–15)^ have been reported. Several *in vitro* studies have demonstrated oleuropein to have higher antioxidant activity than a hydro-soluble analog of tocopherol and inhibitory activity against the proliferation and migration of various cancer cell lines.^(16,17)^

Oleuropein is also known to prevent the progression of hepatic steatosis.^(18–20)^ Poudyal *et al.*^(21)^ reported that olive leaf extracts contain polyphenols such as oleuropein and its major metabolite, hydroxytyrosol, which ameliorate the cardiovascular, hepatic, and metabolic symptoms in rat models with obesity and diabetes induced by high-carbohydrate and high-fat diets (HFD). Olive leaf extracts were reported to lower blood cholesterol^(22)^ and lipid concentrations^(23)^ in cholesterol-fed rats.

Oleuropein attenuates visceral adiposity in mice with HFD-induced obesity^(24)^ and reverses the HFD-induced elevations of adipogenesis-related gene expression.^(24)^ Oi-Kano *et al.*^(25)^ suggested that oleuropein enhanced thermogenesis by increasing the level of the uncoupling protein 1 (UCP1) in interscapular brown adipose tissue and secretion of noradrenalin and adrenaline. Oleuropein was also reported to inhibit the differentiation of 3T3-L1 adipocytes.^(24,26,27)^

In diabetes studies, oleuropein is known to decrease blood glucose level in alloxan-induced diabetic rats^(28)^ and rabbits.^(29)^ Recently, Murotomi *et al.*^(30)^ used the Tsumura Suzuki Obese Diabetes mouse as a diabetic phenotype of type 2 diabetes phenotype and showed that the oleuropein rich diet attenuated hyperglycemia and impaired glucose tolerance without any effect on obesity. However, its effect has not been fully documented for type 2 diabetes caused by obesity.

Therefore, to determine the effect of oleuropein on the glucose tolerance caused in type 2 diabetes and obesity, its effects in skeletal muscle under the lipotoxic condition were examined using C2C12 cells and gastrocnemius muscles of mice fed a high fat diet.

## Materials and Methods

### Materials

Oleuropein (98.3% purity) was supplied by Eisai Food & Chemical Co., LTD. (Tokyo, Japan). Mouse myoblast C2C12 cells were purchased from the Riken BioResource Center (RBC, Tsukuba, Japan). Insulin (Humalin^®^ 100 IU/ml) was purchased from Eli Lily Japan Co. Ltd. (Kobe, Japan) and dissolved in saline for use.

### Cell culture

C2C12 cells were grown in Dulbecco’s Modified Eagle Medium (DMEM, Nissui Co., Ltd., Japan) containing 10% fetal bovine serum (FBS, Biowest, Nuaillé, France) until reaching confluence. Cells were differentiated into myotube cells by incubation in differentiation medium containing 2% horse serum (Invitorogen, Carlsbad, CA).

### Measurement of glucose uptake levels

C2C12 cells were seeded into 6 well plate at 1.5 × 10^6^/well. After five days from the start of differentiation, oleuropein was added to C2C12 myotube cells at the final concentrations of 1, 10 and 100 µM and incubated for 60 min. After washing with phosphate buffer saline (PBS) twice, the culture medium was changed to Krebs-Ringer bicarbonate buffer (KRBB). Cells were pre-incubated for 40 min, and further incubated with 20 µM 2-[^3^H] deoxy-d-glucose (2 kBq/ml) for 30 min. Cell lysate radioactivity was measured using a scintillation counter (Beckman Coulter, Brea, CA), and the amount of uptake was adjusted by protein concentration measured by Bradford assay (Bio-rad, Hercules, CA). Glucose incorporation into C2C12 was also evaluated by calculating the decrease in glucose concentrations in the medium after 4-h incubation that was measured by the method of Miller.^(31)^ To evaluate the effect under the lipotoxic condition, C2C12 cells were incubated with 250 µM palmitic acid.

### Measurement of mRNA expression levels

RNA was extracted using TRIZOL reagent (Invitrogen) from cells of each well. Expression levels of glucose transporter 4 (GLUT4) mRNA were measured by real-time RT-PCR with SYBR Green PCR Master Mix (Applied Biosystems, Foster City, CA). The primer sequences used in this study were as follows: GLUT4, sense 5'-TGA CGC ACT AGC TGA GCT GAA-3'; antisense 5'-AGG AGC TGG AGC AAG GAC ATT-3'.

### Western blot analysis

After pre-incubation with FBS-free DMEM for 3 h, C2C12 cells in 10 mm petri dish were incubated with oleuropein or 10 µM insulin for 2 h. Cell lysate was prepared by a lysis buffer containing 1% Triton X-100 and protease inhibitor (Sigma-Aldrich, St. Louis, MO). The plasma membrane was isolated by ultracentrifugation and sucrose gradient according to the method described by Clancy *et al.*^(32)^ Samples were loaded onto 9% SDS-PAGE gels and transferred onto polyvinylidene difluoride. membranes. The membranes were blocked with a blocking buffer (Blocking One, Nakarai tesque, Kyoto, Japan) for 20 min at 20°C and then incubated overnight with primary antibodies at 4°C. The following primary antibodies were used: GLUT4 (Merk Milipore, Darmstadt, Germany), Akt, phosphor Akt (Ser473), α-AMPK, α-phospho AMPK (Thr 172) and NA^+^/K^+^-ATPase for detection of isolated plasma membrane (Cell Signaling Technology, Danvers, MA).

The membranes were incubated with the corresponding HRP-conjugated secondary antibody (Santa Cruz Biotechnology, Dallas, TX) for 60 min at 20°C. Blots were developed using an enhanced chemiluminescence (ECL) detection kit according to the manufacturer’s instructions (Amersham, Buckinghamshire, UK). Signals were detected by LAS-4000 (Fujifilm Co., Ltd., Tokyo, Japan) and analyzed using Multi Gauge software (Fujifilm Co., Ltd.).

### Animal experiment

Six-week-old male C57BL/6J mice were purchased from CLEA Japan, Inc. (Tokyo, Japan). After pre-feeding a commercial diet (CE-2; CLEA Japan, Inc.) for seven days, the mice were divided into three groups (*n* = 6) and fed experimental diet based on AIN-93G for 12 weeks. Experimental diets were normal-fat diet (NFD; commercial chow containing 13% energy from fat, CE-2 from Clea Japan, Inc.), high-fat diet (HFD, 50% energy from fat), and HFD containing 0.038% oleuropein (OLE), which is the same concentration as previous study^(30)^ used OPIACE (35% oleuropein, 54% saccharide (dextrin) as a stabilizer, 7% other phenolic compounds and others). Composition of the experimental diet was shown in Table 1.

Oral glucose tolerance tests (OGTT) were carried out in the 11th week. After 12 h of fasting, 0.1 ml of 15% glucose/g body weight was orally administered, and the blood drawn from the tail was used to measure glucose concentrations at 0, 15, 30, 90 and 120 min using a blood glucose monitor (ascensia auto disc censor, Byer Yakuhin Ltd., Osaka, Japan). Blood was collected for the insulin tolerance test (ITT) at 0, 10, 20, 30, 60, 90 and 120 min after peritoneal injection of insulin (0.8 µU/g body weight) into the mice, and the glucose level for each sample was measured.

At the end of the experiment period (12 weeks), an aliquot of blood was collected from the tails of mice to measure the levels of fasting glucose and insulin. And then, the blood and gastrocnemius muscles of each mice were collected after 10 min of oral glucose administration (0.1 ml of 15% glucose/g body weight). The concentration of plasma insulin was measured by ELISA kit (Morinaga Institute of Bioscience Inc. Yokohama, Japan). Non-esterified fatty acid was also evaluated using an NEFA kit (Wako Pure Chemical Industries, Osaka, Japan). The Animal Ethics Committee of Ochanomizu University approved all procedures.

### Fluorescence immunohistochemistry

The gastrocnemius muscles of three mice in each group were fixed with 4% paraformaldehyde in PBS. The cryostat sections (10 µm thickness) were incubated with goat anti-GLUT4 antiserum (1:100, G2011, Santa Cruz), and the FITC-labeled secondary antiserum (1:200, J2609, Santa Cruz) was used for fluorescent detection and mounted in ProLong Gold with 4',6-diamidino-2-phenylindole (DAPI, Invitrogen). All confocal images were collected with a confocal laser scanning microscope (LSM710, Carl Zeiss, Oberkochen, Germany). The figures were compiled using Photoshop CS software (Adobe Systems, San Jose, CA).

### Statistical analysis

The results were expressed as mean ± SD. Statistical analysis was performed using analysis of variance (ANOVA) followed by Tukey-Kramer’s test for multiple comparisons. Differences with a probability of 5% or less were considered significant.

## Results

### Oleuropein-induced glucose uptake in C2C12 myotube cells by induction of GLUT4

**Fig. 2:**
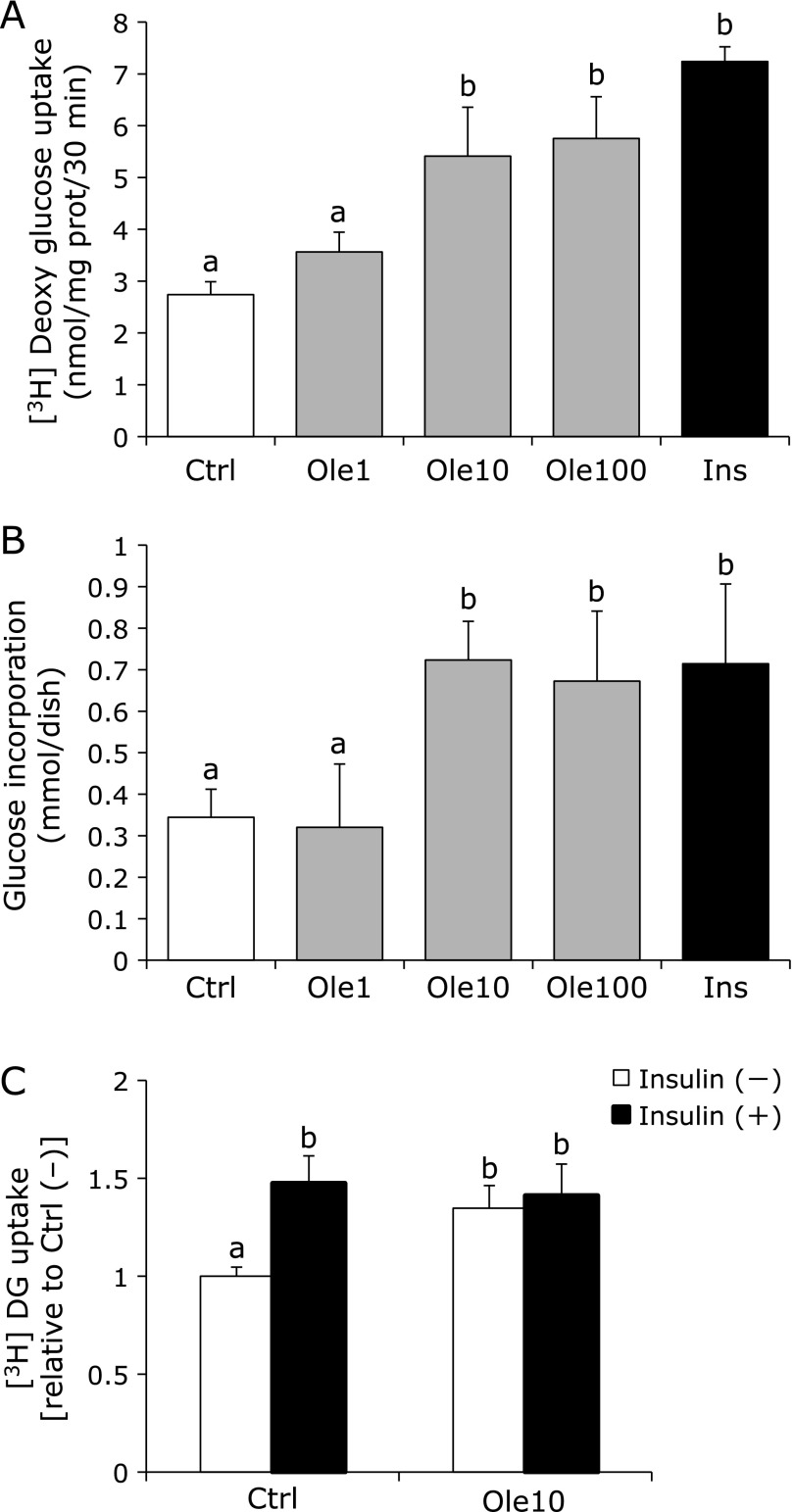
Oleuropein enhanced glucose uptake into C2C12 cells. C2C12 myotube cells were pre-incubated with oleuropein (0, 1,10 and 100 µM; Ctrl, Ole1, Ole10, Ole100) or 100 nM insulin (Ins) for 60 min and then washed with PBS. After pre-incubation with KRBB buffer for 40 min, cells were incubated with 20 µM 2-[^3^H] deoxy-d-glucose (2 kBq/mL) for 30 min. Radioactivity of cell lysate was measured and uptake was adjusted by protein concentration (A). Amounts of glucose incorporated into cells were calculated form the decrease in glucose of medium after incubation for 24 h (B). Glucose uptake was measured with or without insulin (C). All the values were expressed as mean ± SD (*n* = 3). Values not sharing a common roman letter are significantly different (*p*<0.05).

Figure 2A shows the levels of ^3^[H]-deoxy-glucose uptake into C2C12 for 30 min in the presence of 1, 10 and 100 µM oleuropein. Oleuropein (10 µM) was found to be sufficient to enhance the uptake of ^3^[H]-deoxy-glucose by 1.4-fold, the similar as insulin. We also evaluated the glucose incorporation into cells by measurement of decrease in concentrations of glucose in medium for 4-h incubation (Fig. 2B), and confirmed the similar results. No further induction by oleuropein in the presence of insulin was observed (Fig. 2C).

**Fig. 3:**
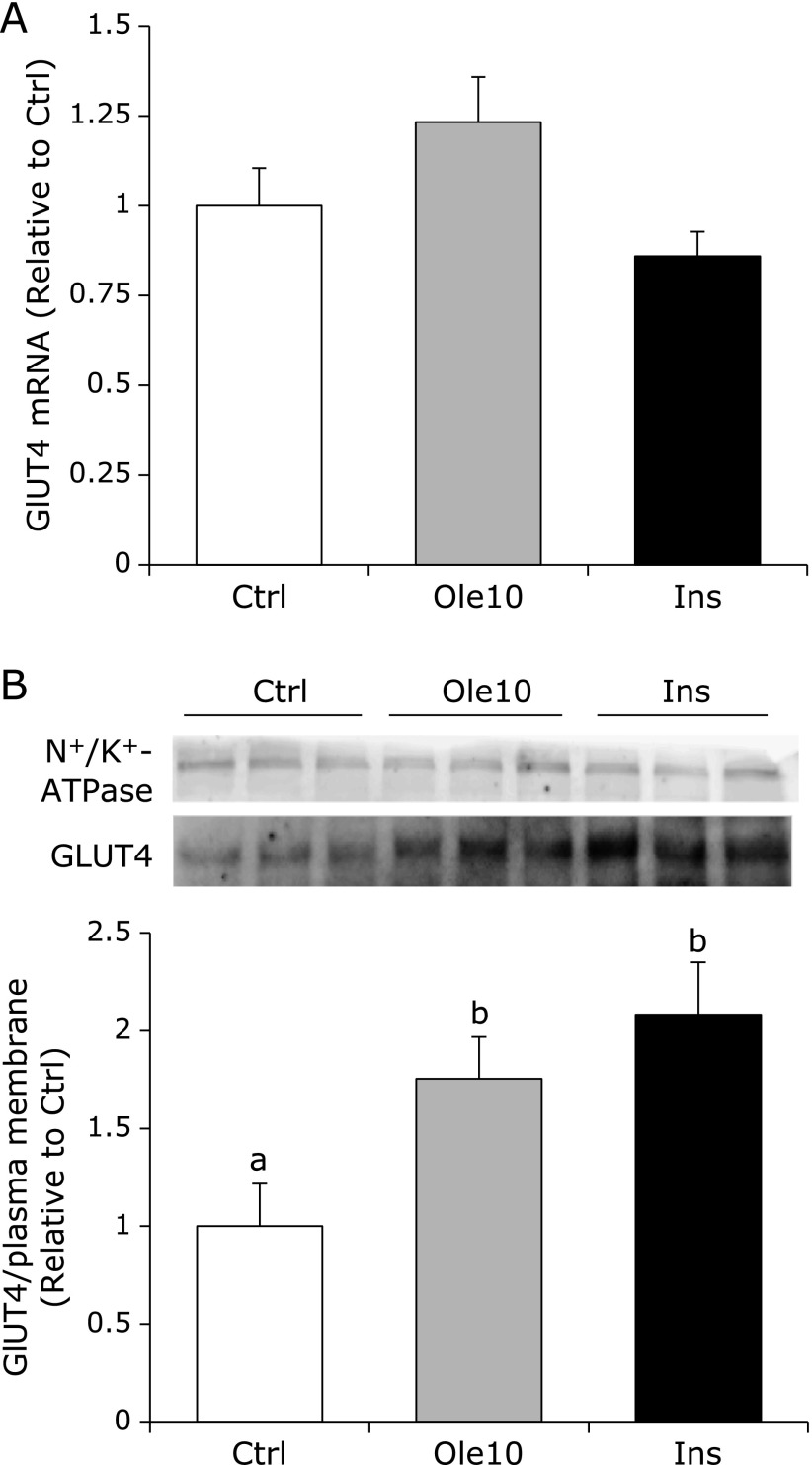
Oleuropein enhanced GLUT4 translocation in plasma membrane. GLUT4 mRNA expression was measured by a real-time RT-PCR (A). GLUT4 expression in the plasma membrane of C2C12 cells were analyzed by western blotting, and normalized by the plasma membrane protein detected by N^+^/K^+^ ATPase antibody (B). Values are given as mean ± SD (*n* = 3). Values not sharing a common roman letter are significantly different (*p*<0.05).

As shown in Fig. 3, expression levels of GLUT4 mRNA in C2C12 cells were not significant differences between control and oleuropein treatment, however, Western blot analysis revealed oleuropein to have induced a 1.8-fold increase in the expression of GLUT4 protein in C2C12 plasma membrane, whereas insulin increased by two-fold. These results suggest that oleuropein induces the amounts of GLUT4 protein into the cell surface and consequently increases the glucose uptake.

### Effect of oleuropein on signal transduction to promote GLUT4 translocation

**Fig. 4:**
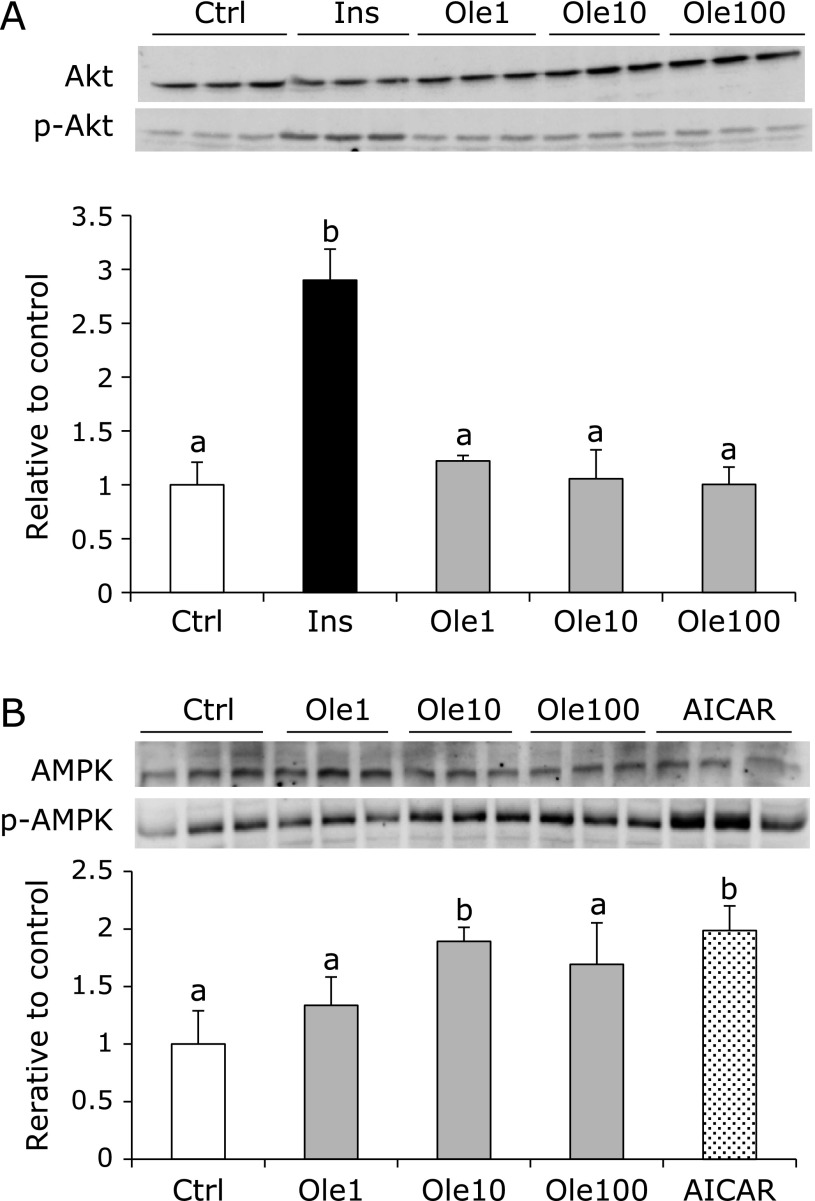
Oleuropein activated the phosphorylation of AMPK but not Akt. Lysate from cells treated with oleuropein (1, 10 and 100 µM; Ctrl, Ole1, Ole10, Ole100) or 100 nM insulin (Ins) for 20 min were analyzed by western blotting using Akt and phospho-Akt (Ser 473) antibody (A). Lysate from cells treated with oleuropein (1, 10 and 100 µM) or AICAR (500 µM) for 24 h and blotted by AMPK and phospho-AMPK (Thr 172) antibody (B). Values are given as mean ± SD (*n* = 3). Values not sharing a common roman letter are significantly different (*p*<0.05).

As oleuropein induces glucose uptake, the effect on the phosphorylation of Akt, which is downstream of the insulin signal, was investigated to elucidate its mechanism. As shown in Fig. 4A, insulin activates Akt significantly, whereas oleuropein does not increase the phosphorylation of Akt despite results showing 10 µM oleuropein to have an effect equivalent to that of 100 nM insulin in inducing glucose uptake (Fig. 4A). AMPK phosphorylation, another signaling pathway was also examined. Figure 4B shows that oleuropein activates AMPK phosphorylation significantly at 10 µM, and its effect was found to be similar to that of 500 µM AICAR, which is the AMPK activator.

### Oleuropein improved lipotoxicity of C2C12 induced by palmitic acid

**Fig. 5:**
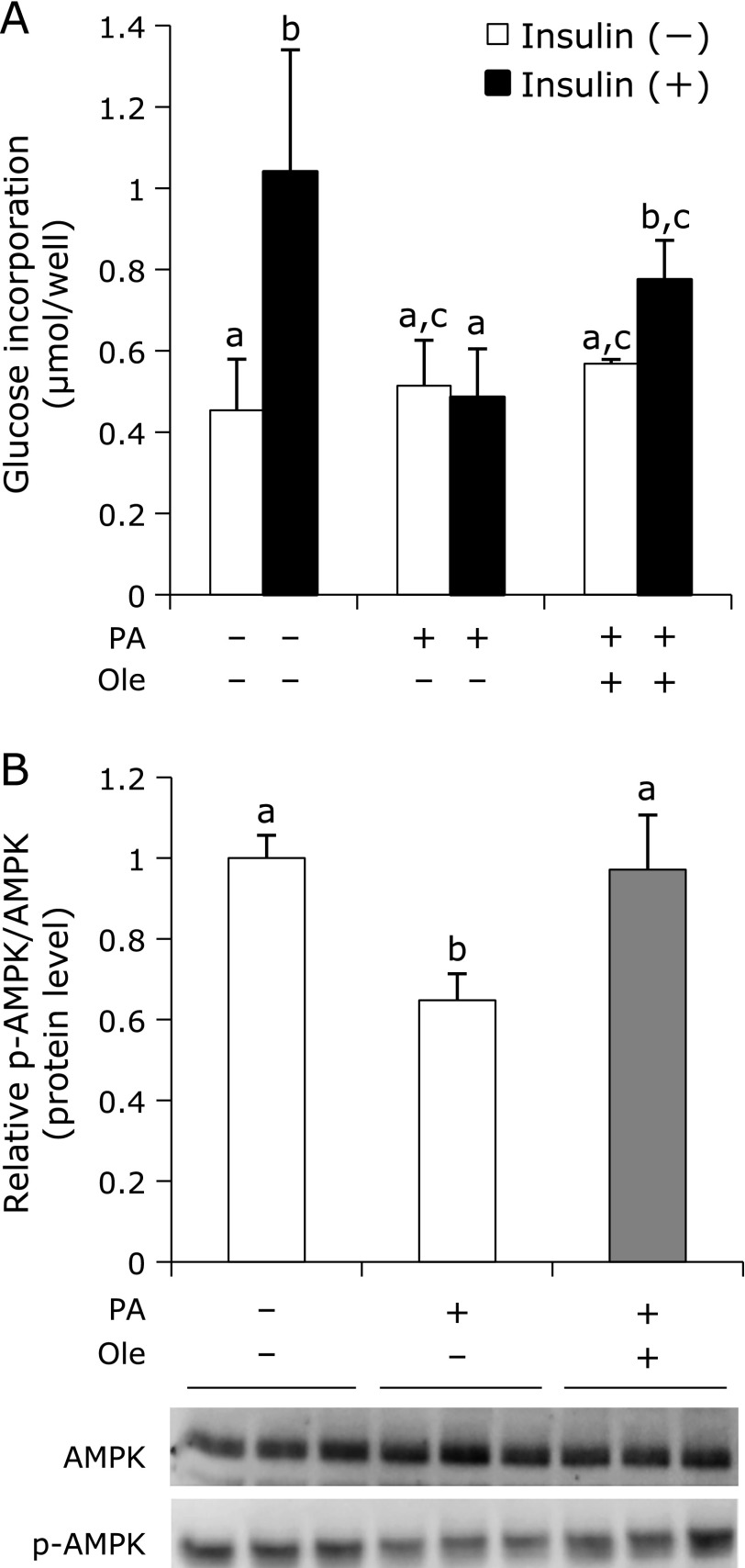
Oleuropein stimulates AMPK activation and improves insulin resistance in the presence of palmitic acid. C2C12 cells were pre-incubated with 10 µM oleuropein (Ole) and/or 250 µM palmitic acid (PA), the levels of glucose incorporation for 24 h were measured with or without insulin (A). C2C12 cells were incubated with 10 µM oleuropein and/or 250 µM palmitic acid for 24 h followed by western blotting (B). Values are expressed as mean ± SD (*n* = 3). Values not sharing a common roman letter are significantly different (*p*<0.05).

The amount of cellular incorporation of glucose was evaluated in C2C12 cells with 250 µM palmitic acid as the model of insulin resistance induced by obesity (Fig. 5A). As shown in Fig. 5A, addition of insulin enhanced glucose incorporation into C2C12 cells by 2.5-fold whereas palmitic acid inhibited the increase by insulin; however, oleuropein recovered the glucose incorporation in the presence of insulin. It suggested that oleuropein improved insulin resistance of C2C12 cells. Although palmitic acid decreased the phosphorylation of AMPK in C2C12 significantly, oleuropein was observed to enhance the phosphorylation of AMPK in the presence of 250 µM palmitic acid (Fig. 5B).

### Oleuropein enhanced GLUT4 translocation *in vivo*

 To determine whether the effect of oleuropein on skeletal muscle would be reproducible *in vivo*. High fat feeding increased the body weights and white adipose tissues; however, the body weights and white adipose tissue weights of OLE-fed mice were similar to those of HFD-fed mice (Table 2). Average amount food intake of HFD and OLE was 3.87 ± 0.19 g/mouse/week and 3.84 + 0.19 g/mouse/week, respectively. Oleuropein supplementation was not shown any adverse effect. Results of OGTT were expressed as AUC (area under curve) in Table 2, showing no significant changes between OLE and HFD. In OLE-fed mice, fasting blood glucose levels were lower than HDF mice. Insulin resistance evaluated as HOMA-IR was significantly improved in OLE-fed mice compared to HFD-fed mice (Table 2).

**Fig. 6:**
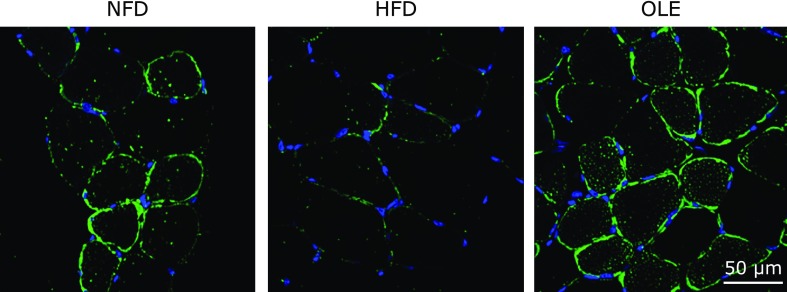
Oleuropein induced GLUT 4 translocation in skeletal muscle of mice with HFD-induced obesity. Representative confocal fluorescence images with anti-GLUT4 antiserum in NFD, HFD, and OLE groups. FITC-labeled secondary antibody was used to detect GLUT4 (green), and nuclei were counterstained with 4',6-diamidino-2-phenylindole (DAPI) (blue).

Figure 6 showed the GLUT4 protein localization in gastrocnemius muscles by immunohistochemistry using a confocal laser scanning microscope. GLUT4 signals in the HFD group were localized in both cell membrane and cytoplasm, they were weakly detected in some cells. In the OLE group, on the contrary, GLUT4 signals were more frequently observed in almost all muscle cells. GLUT4 was mainly distributed in the cell membrane and several vesicular signals were also detected in the cytoplasm. It suggested that orally ingested oleuropein promoted GLUT4 membrane translocation in the gastrocnemius muscle under the lipotoxic condition.

## Discussion

In this study, we investigated the effect of oleuropein on the skeletal muscle, which is important for glucose uptake and for maintaining normal blood glucose levels. As shown in Fig. 2, 10 µM oleuropein was sufficient to enhance glucose uptake in C2C12 cells, by inducing AMPK signaling, but not Akt. Oleuropein did not show the synergistic effect with insulin (Fig. 2C), suggesting that the regulation of GLUT4 by AMPK signaling is independent from insulin signal. Similar results were reported recently by Hadrich *et al.*,^(33)^ who assessed the phosphorylation of AMPK, Akt, IRS (insulin receptor substrate), and GLUT4 at oleuropein concentrations of 200–400 µM. However, we used much lower concentration (10 µM) and demonstrated it to be sufficient to express beneficial effects on C2C12 cells. Kano *et al.*^(34)^ reported that 10 µM oleuropein was detected in plasma of rat after 240 min of oral administration (10 mg/kg). Therefore the concentration of oleuropein we used in this study is supposed to be physiological condition.

Hadrich *et al.*^(33)^ also showed that reactive oxygen species (ROS) produced by hydrogen peroxide decreased on pre-treatment with oleuropein, and suggested that oleuropein activated AMPK phosphorylation via its anti-oxidative function. Oxidative stress is generally believed to be a mediator of insulin resistance, because hyperglycemia has been implicated in the activation of several major signal transduction pathways, such as NFκB, JNK/SAPKs, and p38 MAPK.^(35,36)^ However, the mechanism of activation of AMPK by oleuropein is still unclear. Sato *et al.*^(37)^ demonstrated the possibility of oleuropein and oleanoic acid acting as agonists for transmembrane G protein-coupled receptor 5 (TGR5), a receptor activated by bile acid and supposed that be involved in the anti-diabetic effect of olive oil. Some studies suggest that oleuropein inhibits PPARγ expression.^(26,27)^ Further study on the molecular target of oleuropein is essential.

Oleuropein is known to partially metabolize to tyrsol and hydroxyltyrosol.^(38)^ These metabolites possess stronger anti-oxidative activity than oleuropein.^(39)^ Although we did not compare the effect of oleuropein with that of its metabolites, oleuropein itself was clearly shown to induce GLUT4 translocation to the cell membrane and glucose uptake into the cells.

Lipotoxicity, characterized by a high concentration of non-esterified fatty acid and induced especially by palmitic acid in blood, aggravates not only insulin tolerance and beta cell function, but also whole body inflammation in type 2 diabetes and metabolic syndrome caused by obesity.^(40)^ Therefore, C2C12 cells treated with palmitic acid were used in this study as a model of lipotoxicity and insulin resistance caused by obesity. As shown in Fig. 5, oleuropein improved insulin sensitivity in C2C12 cells treated with high concentrations of palmitic acid. Toll-like receptor 4 (TLR4) is well known to link to high fat induced insulin resistance.^(41,42)^ TLR2 is thought to be essential for insulin resistance induced by palmitic acid in C2C12 cells,^(43)^ however, it is not clearly known whether AMPK and TLRs are connected. Although we measured the mRNA expression levels of IL6 (interleukin 6), cytokine in down-stream of the TLR4 signals, treatment of oleuropein did not change the levels significantly between with and without palmitic acid (data not shown). As we could not clarify the details how oleuropein improves insulin resistance in the presence of palmitic acid in this study, so far, further studies will be needed.

Although the effect of oleuropein on type 1 diabetes using rats^(28)^ and rabbits^(29)^ administered with alloxan has already been reported to protect the liver and serum from oxidative stress,^(28)^ its effect on type 2 diabetes mellitus caused by obesity has not yet been fully understood.

Some studies have demonstrated the anti-obesity and anti-hypolipidemic effects of oleuropein.^(21,24)^ In Wistar rats fed a high-carbohydrate and HFD for 16 weeks, treatment with olive leaf extracts including oleuropein and hydroxytyrosol reduced abdominal fat circumference and fat pads without changing body weights in either of the two groups.^(21)^ Oi-Kano *et al.*^(25,44)^ reported oleuropein-activated thermogenesis through UCP1 expression and beta adrenalin receptors. In our study, OLE-fed mice showed no significant difference in body weights compared HFD-fed mice, however, immuno histochemical analysis clearly showed that oral intake of oleuropein promoted GLUT4 translocation in skeletal muscle as shown by results in C2C12 cells.

Human study that evaluated olive oil or Mediterranean diet demonstrated its beneficial effects on cardiovascular health and metabolic syndrome.^(45,46)^ The data examining the effect of olive polyphenols on glucose homeostasis are limited; however, de Bock *et al.*^(47)^ reported the effectiveness of oleuropein against type 2 diabetes mellitus induced by obesity in a randomized controlled study in humans. We suggest that one of the mechanisms of oleuropein might be improvement of insulin sensitivity in skeletal muscle by stimulation of GLUT4 translocation independently of insulin signals.

## Figures and Tables

**Table 1:** Composition of the experimental diets

Ingredients (g)	HFD	OLE
β-Corn starch	112.4	112.4
α-Corn starch	37.5	37.5
Sucrose	300.0	300.0
Milk casein	200.0	200.0
Soybean oil	30.0	30.0
Lard	220.0	220.0
Cellulose powder	50.0	50.0
Mineral mix (AIN-93G)	35.0	35.0
Vitamin mix (AIN-93G)	10.0	10.0
l-Cystine	3.0	3.0
Choline hydrogen tetrate	2.5	2.5
Oleuropein	0.0	0.38

Both of soybean oil and lard contains 0.05% (w/w) *t*-butylhydroquinone.

**Table 2:** Changes in body and fat tissue weight and blood markers related to insulin resistance after 12 weeks for each experimental diets

	NFD (CE2)	HFD	OLE
Final body weight (g)	29.8 ± 2.5^a^	43.2 ± 1.5^b^	40.1 ± 3.3^b^
White adipose tissue (g)	0.56 ± 0.11^a^	1.98 ± 0.31^b^	1.81 ± 0.73^b^
Brown adipose tissue (g)	0.14 ± 0.06^a^	0.36 ± 0.71^b^	0.29 ± 0.08^b^
Fasting blood glucose (mg/dl)	89.7 ± 2.0^a^	146.7 ± 12.3^b^	122.0 ± 15.8^c^
Fasting insulin (pg/dl)	0.91 ± 0.10^a^	3.29 ± 1.03^b^	2.72 ± 0.95^b^
NEFA (µE/L)	150 ± 2.46^a^	158 ± 2.35^a^	144 ± 1.98^a^
AUC (glucose g/dl)	31.8 ± 2.57^a^	47.4 ± 7.86^b^	41.8 ± 4.05^b^
HOMA-IR	0.152 ± 0.03^a^	1.07 ± 0.33^b^	0.442 ± 0.14^a^

Mice were fed by NFD (normal fat diet), HFD (high fat diet) and OLE (HFD containing 0.038% oleuropein) for 12 weeks. AUC was calculated from the blood glucose levels obtained by OGTT at 0, 15, 30, 90 and 120 min after oral administration of glucose. HOMA-IR was expressed the value calculated as follows; (fasting glucose levels × fasting insulin levels)/405. Values are expressed as mean ± SD (*n* = 6). Significant differences were analyzed by Turkey-Kramer. Values not sharing a common roman letter are significantly different (*p*<0.05).

## Abbreviations

Akt: protein kinase B
AMPK: adenosine monophosphate-activated protein kinase
ANOVA: analysis of variance
ECL: enhanced chemiluminescence
FITC: fluorescein isothiocyanate
GLUT4: glucose transporter 4
HFD: high-fat diet
HRP: horse radish peroxidase
IL6: interleukin 6
IRS: insulin receptor substrate
KRBB: Krebs-Ringer bicarbonate buffer
NFD: normal fat diet
OLE: oleuropein diet
PBS: phosphate buffer saline
ROS: reactive oxygen species
SDS-PAGE: sodium dodecyl sulfate poly-acrylamide gel electrophoresis
TLR4: Toll-like receptor 4
UCP1: uncoupling protein 1
